# DeepLabV3+/Efficientnet Hybrid Network-Based Scene Area Judgment for the Mars Unmanned Vehicle System

**DOI:** 10.3390/s21238136

**Published:** 2021-12-05

**Authors:** Shuang Hu, Jin Liu, Zhiwei Kang

**Affiliations:** 1 College of Information Science and Engineering, Wuhan University of Science and Technology, Wuhan 430081, China; 201904907142@wust.edu.cn; 2 College of Computer Science and Electronic Engineering, Hunan University, Changsha 410082, China; jt_zwkang@hnu.edu.cn

**Keywords:** hybrid neural network, UAV, feature extraction, scene area judgment

## Abstract

Due to the complexity and danger of Mars’s environment, traditional Mars unmanned ground vehicles cannot efficiently perform Mars exploration missions. To solve this problem, the DeepLabV3+/Efficientnet hybrid network is proposed and applied to the scene area judgment for the Mars unmanned vehicle system. Firstly, DeepLabV3+ is used to extract the feature information of the Mars image due to its high accuracy. Then, the feature information is used as the input for Efficientnet, and the categories of scene areas are obtained, including safe area, report area, and dangerous area. Finally, according to three categories, the Mars unmanned vehicle system performs three operations: pass, report, and send. Experimental results show the effectiveness of the DeepLabV3+/Efficientnet hybrid network in the scene area judgment. Compared with the Efficientnet network, the accuracy of the DeepLabV3+/Efficientnet hybrid network is improved by approximately 18% and reaches 99.84%, which ensures the safety of the exploration mission for the Mars unmanned vehicle system.

## 1. Introduction

In the solar system, Mars exploration is particularly important. However, the insufficiency of human knowledge on Mars seriously limits the technological development of Mars exploration [1]. In the past 20 years, Mars exploration missions have been implemented, such as the MESUR Pathfinder in 1996, the Mars Global Surveyor in 1996, the Mars Odyssey in 2001, the Mars Exploration Rover in 2003, the Mars Reconnaissance Orbiter in 2005, the Phoenix in 2007, and the Curiosity Mars detector in 2011. Three Mars rovers were launched to Mars in the summer of 2020, including NASA’s Mars 2020 vehicle, the European and the Russian Rosalind Franklin Mars vehicle, and the Chinese Mars vehicle TianWen 1. On 15 May 2021, China’s Zhurong Mars unmanned vehicle successfully landed on Mars. These Mars unmanned vehicle systems for Mars exploration have sent back valuable data. Therefore, Mars exploration is an important means for human beings to understand Mars and the universe [2].

Mars ground vehicles are used by all Mars landing projects [3]. It is worth noting that NASA recently used a Mars unmanned aerial vehicle [4] for the first time. On 19 April 2021, NASA officially announced that the first Mars unmanned aerial vehicle successfully completed its first flight in the Jezero impact crater of Mars. The Mars unmanned aerial vehicle carries two cameras. The color camera at the bottom can take high-resolution photos of 13 million pixels, while the navigation camera has a lower resolution of only 500,000 pixels. After the Mars unmanned aerial vehicle has landed [5], the aerial data are transmitted back to Earth by the Mars unmanned ground vehicle. This is also the first time that humans have completed a powered flight in the atmosphere outside the Earth [6]. In this paper, the Mars unmanned vehicle system comprises an unmanned ground vehicle and an unmanned aerial vehicle. One of the major missions of the Mars unmanned vehicle system is to inspect the surface of Mars.

Currently, Mars exploration is full of uncertainties, resulting in the failure of Mars exploration missions. On 22 March 2011, the U.S. rover Spirit fell into quicksand while exploring the surface of Mars. Judgment of the scene area is particularly critical in Mars exploration missions. Therefore, in order to complete the Mars exploration mission, it is necessary to judge the scene area efficiently for safety. Intelligent technology is an effective method to enhance the autonomous ability of the unmanned vehicle system. Deep learning is one such intelligent technology. Due to its high accuracy, deep learning is widely used in unmanned automatic driving and scene area judgment [7,8].

This artificial intelligence technology is applied to Mars exploration missions and improves their efficiency. Simonyan [9] proposed a VGG (Visual Geometry Group) network, which solves the problem of deep network training. It is widely used in unmanned driving and area recognition. However, the Resnet network affects network convergence when the gradient explodes. Huang [10] proposed a Densenet network, which solves the problem that feature information cannot be recycled through dense network layer connections. Due to the large number of parameters in Resnet and Densenet, training takes a long time. Chen [11] proposed an Addernet (Addition) network, which solves the problem of a large amount of calculation. In recent years, researchers proposed lightweight networks to solve the problem of large parameters and time consumption. Sandler [12] proposed a lightweight MobilenetV2 network that solves the problems of multi-parameters and long time consumption. Tan [13] proposed an Efficientnet network with fast reasoning speed, high accuracy, and few parameters. In particular, this network has high accuracy in unmanned fields, such as scene area judgment, classification, and it can also transmit information well. Compared with Resnet, Densenet, Addernet, and MobilenetV2 networks, the Efficientnet network has higher accuracy and fewer parameters. However, due to the low number of Efficientnet parameters, when the amount of extracted feature information is large, accuracy decreases. Therefore, Chen [14] proposed a DeepLabV3+ network to solve the problem of extracting a large amount of feature information. DeepLabV3+ network uses the method of hole convolution and enlargement of the receptive field to extract images with a large amount of feature information, which ensures the effective acquisition such information.

In this paper, in order to improve accuracy, the DeepLabV3+ network is combined with the Efficientnet network. A DeepLabV3+/Efficientnet hybrid network is proposed for scene area judgment. The scene areas are divided into three categories, including safe area, report area, and dangerous area. The categories are notified to the Mars unmanned vehicle system, and the Mars unmanned vehicle system performs three operations: (1) safe area, the Mars unmanned ground vehicle continues to explore; (2) report area, DeepLabV3+/Efficientnet hybrid network saves the results, and the Mars unmanned ground vehicle performs deceleration; and (3) dangerous area, the Mars unmanned vehicle system sends the Mars unmanned aerial vehicle exploring.

This paper is organized into five sections. After the introduction, Section 2 describes the process of the overall framework for the Mars unmanned vehicle system. In Section 3, the DeepLabV3+/Efficientnet hybrid network is proposed. In Section 4, experimental results are shown. Conclusions are drawn in Section 5.

## 2. Description

**Figure 1:**
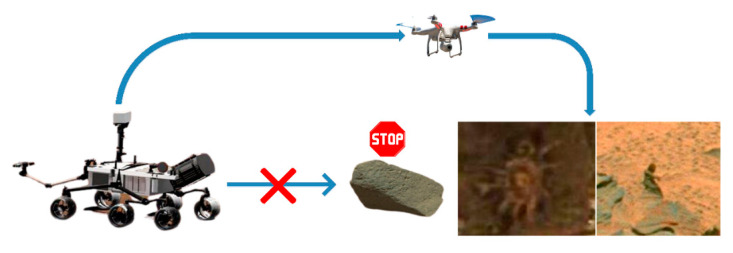
Schematic of the Mars unmanned vehicle system.

In Section 2, the Mars unmanned vehicle system and the overall process framework are introduced as follows:

1. Mars unmanned vehicle system. Due to the restriction of the harsh environment of Mars, the Mars unmanned ground vehicle is unable to reach the designated position. Therefore, the Mars unmanned vehicle system is conceived. The Mars unmanned vehicle system is composed of two parts: the Mars unmanned ground vehicle and the Mars unmanned aerial vehicle. The Mars unmanned vehicle system is equipped with artificial intelligence algorithms. The schematic of the Mars unmanned vehicle system is shown in Figure 1. When the Mars unmanned ground vehicle encounters obstacles, it cannot pass through them, thus failing to move forward. At this time, the Mars unmanned vehicle system launches the Mars unmanned aerial vehicle to bypass obstacles and discover interesting objects.

2. Feature extraction. The image taken by the camera is entered into the DeepLabV3+ network to extract image features. The feature is used as the input of the Efficientnet network to judge the scene area.
3. Scene area judgment. The output of the Efficientnet network is divided into three categories: safe area, report area, and dangerous area. Correspondingly, the Mars unmanned vehicle system performs pass, report, and send, respectively.

**Figure 2:**
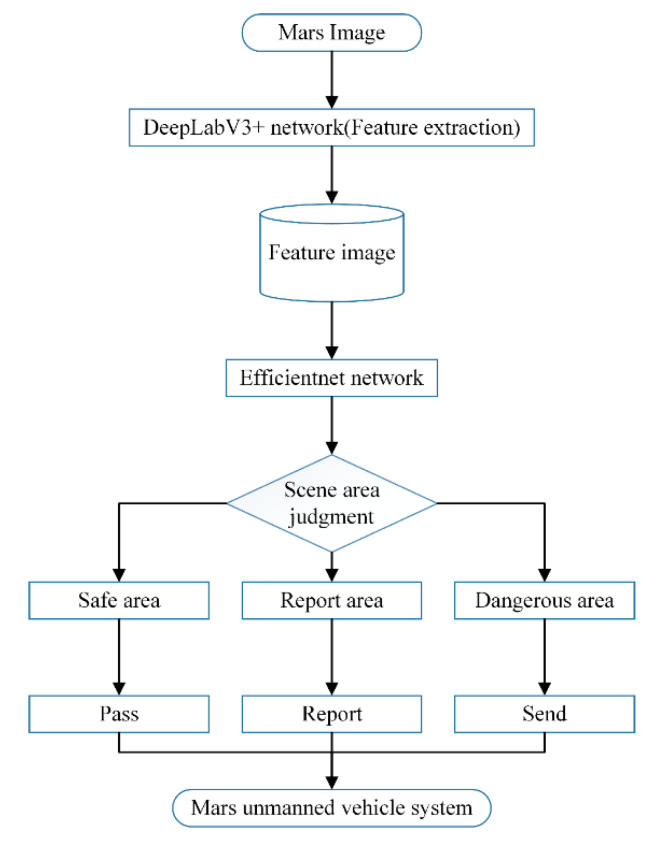
Process of the overall framework.

The process of the overall framework is shown in Figure 2.

## 3. DeepLabV3+/Efficientnet Hybrid Network

In Section 3.1 and Section 3.2, the DeepLabV3+ network and the Efficientnet network are introduced. In Section 3.3, the process of the DeepLabV3+/Efficientnet hybrid network is given.

### 3.1. DeepLabV3+

#### 3.1.1. Structure Network Model of DeepLabV3+

Semantic segmentation is an important technical method for feature extraction [15]. In particular, DeepLab models are widely used in feature extraction. They can extract each pixel point in the image and obtain the feature information of the target. Since 2014, Chen [16,17,18] successively proposed DeepLabV1, DeepLabV2, DeepLabV3, and DeepLabV3+ models. The DeepLabV3+ network model introduces a common encoder–decoder form of semantic segmentation, and it uses the decoder to obtain the information of the encoder. The decoder restores the structure and spatial dimensions of the target image. The encoder uses hole convolution [19] to balance accuracy and time loss.

Compared with PSPnet (Pyramid Scene Parsing Network) [20], FCNnet (Fully Convolutional Network) [21], and Unet [22], the advantage of the DeepLabV3+ network is that it uses hole convolution, which enlarges the receptive field of feature information without a loss of information. On Mars, feature information is extremely critical. In order to allow the DeepLabV3+ network to obtain as much feature information as possible, spatial pyramid pooling [23] is used to achieve multi-scale feature information extraction. Low-level feature information is fused with high-level feature information to restore the key information of the target image.

**Figure 3:**
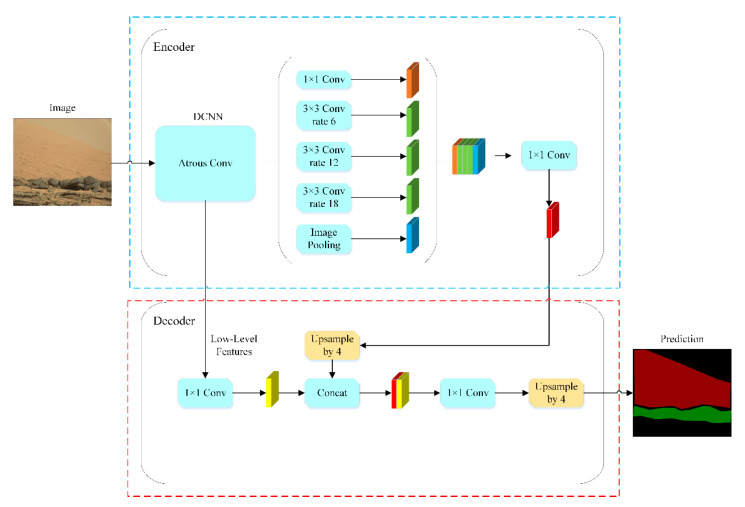
DeepLabV3+ network structure.

**Figure 4:**
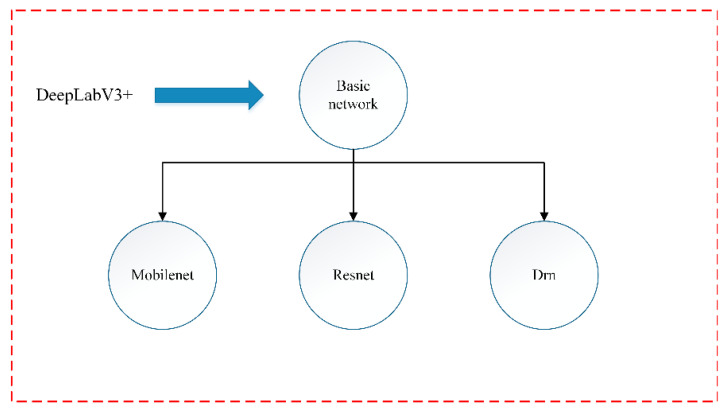
Basic network diagram of DeepLabV3+.

The main network model structure of DeepLabV3+ is shown in Figure 3. Its base network and the hole convolutional space pyramid module together constitute the encoder. The image of Mars is entered into the encoder. The encoder obtains high-level feature information. In addition, high-level feature information is up-sampled four times in the decoder and fused with low-level feature information to obtain the whole feature information of the Mars image. The whole feature information passes through the Softmax classification layer to obtain the segmentation image corresponding to the original image. The basic networks of DeepLabV3+ include Drn (Network of Dual Regression) [24], Resnet (Network of Residual) [25], and Mobilenet (Convolutional Neural Networks for Mobile) [26]. The basic network diagram of DeepLabV3+ is shown in Figure 4.

#### 3.1.2. Implementation Process of DeepLabV3+

The whole implementation process of the DeepLabV3+ network has four steps:

1. The Mars unmanned vehicle system takes an original Mars image and extracts the original Mars image features through the mainstream deep convolutional neural network [27,28] (DCNN, which also adds a hollow convolution) and obtains high-level and low-level semantic features.
2. High-level semantic features are separately convolved and pooled in the hole convolution pyramid module. The module obtains five feature images and connects the five features obtained. The module uses a 1 × 1 convolutional layer to perform convolution operations for a single high-level semantic feature.
3. Low-level semantic features are obtained by the hole convolutional layer. Furthermore, in the decoder, the semantic feature information is operated by the deep convolutional network layer. Low-level and high-level semantic features have the same resolution.
4. Low-level and high-level semantic features are combined and refined through a 3 × 3 convolutional layer. The refined result adopts bilinear up-sampling four times to obtain the image of the feature extraction.

### 3.2. Efficientnet

#### 3.2.1. Structure Network Model of Efficientnet

Traditional convolutional neural networks generally expand the network by adjusting the resolution of the input image, network depth, and the number of convolution channels, while EfficientNet uses the model composite scaling method to perform network expansion. The specific method is to specify the composite co-efficiency while constraining the image resolution, network width, and depth.

**Figure 5:**
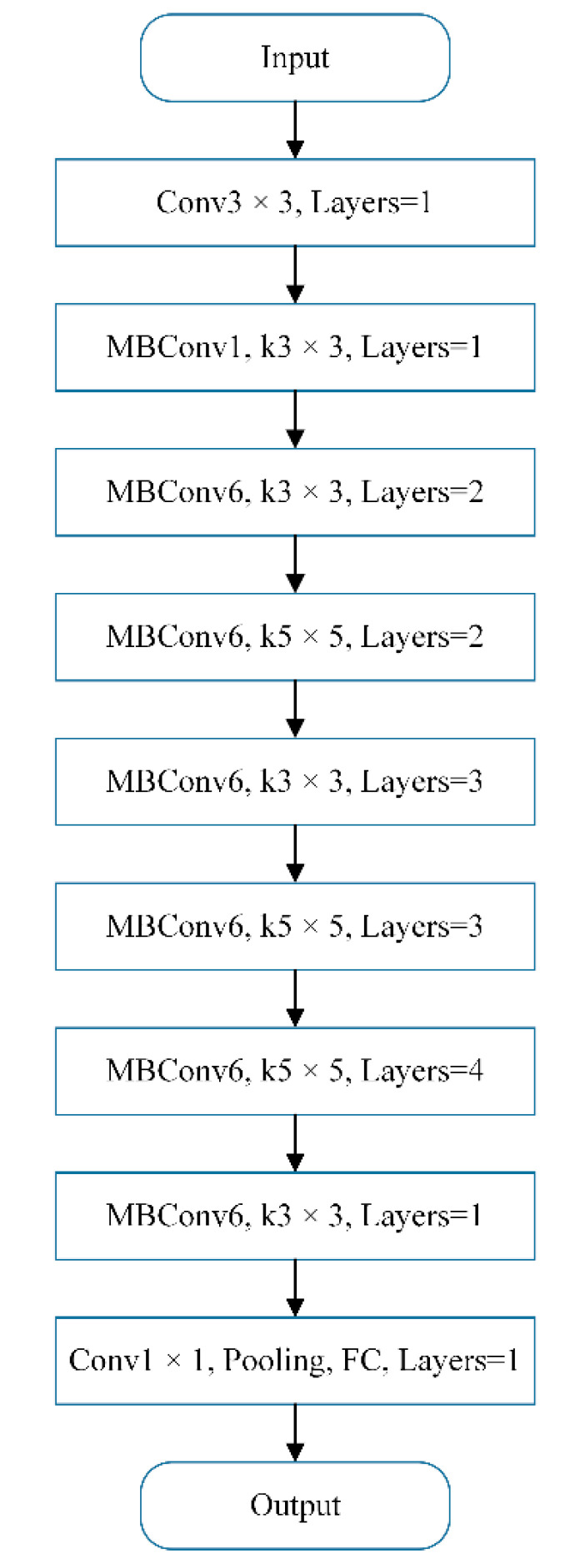
Structure of Efficientnet network.

The main backbone network is constructed by using modules in the MobileNet network. The network flowchart is shown in Figure 5.

The network structure is divided into nine stages in total. The first stage is a normal convolutional layer (including activation function) with a convolution kernel size of 3 × 3. Stage 2~Stage 8 are all repeatedly stacking MBConv (Mobilenet Convolution) structures (the layers in the last column indicate how many times the stage repeats). Stage 9 consists of a common 1 × 1 convolutional layer (including activation function), an average pooling layer, and a fully connected layer. Each MBConv in Figure 5 is followed by a number 1 or 6, where 1 or 6 is the magnification factor.

#### 3.2.2. Implementation Process of Efficientnet

The whole implementation process of the Efficientnet network has three steps:

1. The image is extracted by a 3 × 3 convolutional layer and is entered by multiple block structures to further extract feature information.
2. In order to enhance the ability to express features in high-dimensional space, and avoid the gradient disappearance during model training, ReLU (Rectified Linear Unit) function is used as the activation function of the network. ReLU activation function can accelerate the network convergence and reduce the value of the loss.
3. Efficientnet uses the convolution-pooling-full connection operation to replace the classifier and the Softmax regression function to normalize the full connection layer. Efficientnet realizes the recognition of feature images and classification.

### 3.3. DeepLabV3+/Efficientnet Hybrid Network for Scene Area Judgment

**Figure 6:**
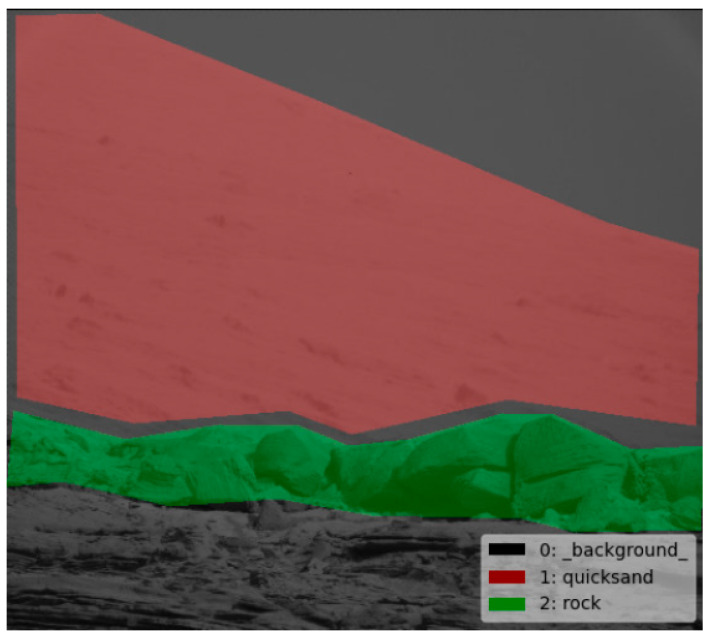
Image of feature extraction.

Mars roads are mainly composed of rocks, quicksand, and other ravines. Therefore, the feature information of Mars includes rocks and quicksand. Mars road image extracts by the DeepLabV3+ model are shown in Figure 6. The feature information is shown in Table 1.

**Figure 7:**
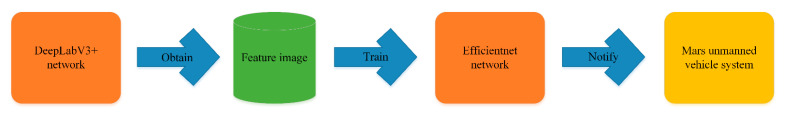
Realization process of hybrid network.

The feature image is entered into scene area judgment, and the output comes from the Efficientnet network. The process of the hybrid network for scene area judgment is shown in Figure 7.

## 4. Experiments

Firstly, the Mars32K dataset is introduced in Section 4.1, and then the experimental process is described in Section 4.2. Finally, experimental results are given in Section 4.3.

### 4.1. Dataset

The Mars32K dataset (Mars32K, https://dominikschmidt.xyz/mars32k/ accessed on 26 November 2018) is obtained by NASA Curiosity on the surface of Mars. NASA Curiosity photographed the dataset and brought it back. The availability of the dataset is reliable. This dataset is used to verify the DeepLabV3+/Efficientnet hybrid network. This dataset is processed from the actual environment of Mars.

This dataset comes from NASA Curiosity. The main feature information of this dataset includes rocks and quicksand. The dataset is manually annotated in order to prevent the model jitter problem caused by the training process. Therefore, the dataset is randomly selected and divided into 480 training sets and 130 validation sets. The process of randomly assigning the dataset can make the model fit well. The dataset distribution is shown in Table 2.

Due to the small number of samples in the dataset, it has the effect of underfitting. Therefore, in order to solve the problem of underfitting, we perform data augmentation [29] processing on the dataset. Data augmentation processing includes the following eight methods: (1) flip transform; (2) random crop; (3) color jittering; (4) translation shift; (5) scale; (6) contrast; (7) noise disturbance; (8) rotation and reflection.

The dataset of data augmentation is shown in Table 3.

The dataset after data augmentation adapts to our model. Data augmentation improves the accuracy of the model.

**Figure 8:**
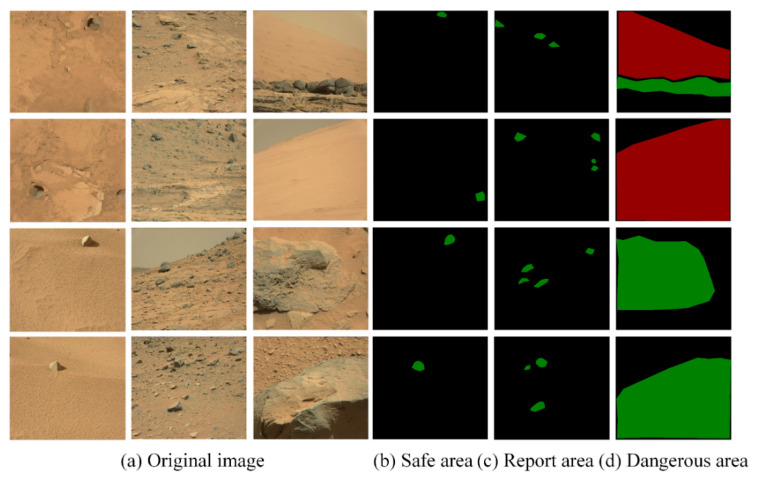
Samples of feature information result image. Three images of the original image (**a**) correspond to the images in (**b**–**d**), respectively.

As mentioned in Section 3.3, the feature information of the Mars image is extracted, and the samples of feature information images are shown in Figure 8.

### 4.2. Experiment Procedures

A scene area judgment based on the DeepLabV3+/Efficientnet hybrid network method is proposed for the Mars unmanned vehicle system. It extracts feature information obtained by the DeepLabV3+ algorithm and obtains the output provided by the Efficientnet network. The Mars unmanned vehicle system is based on intelligent algorithm to control the next exploration of the Mars unmanned ground vehicle and the Mars unmanned aerial vehicle. The DeepLabV3+ network is trained, the corresponding training model is saved, and the output of the scene area judgment is obtained.

In the experimental process, the training round is set to 500 epochs of the DeepLabV3+ algorithm model, and the training round is set to 100 epochs of the Efficientnet algorithm model.

### 4.3. Results

In Section 4.3.1, Section 4.3.2, Section 4.3.3, the experimental results of feature extraction, MIOU, FWIOU, and the scene area judgment are given, respectively. In Section 4.3.4, the comparison result of Efficientnet is given.

#### 4.3.1. Feature Extraction

**Figure 9:**
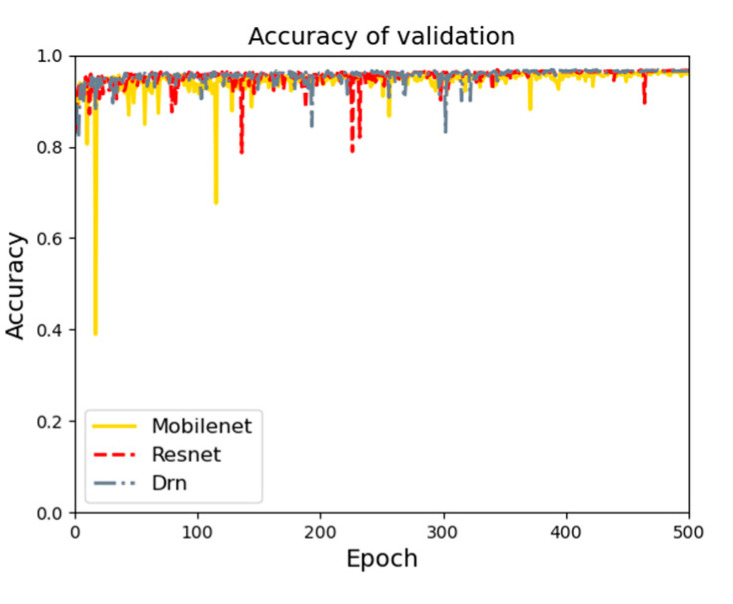
Accuracy curve of feature extraction.

Three base networks are used for feature extraction. Figure 9 shows the feature extraction accuracy curve diagrams using three base networks. On the test set, three base networks are compared, and the Drn base network works best. The accuracy on the test set is 97.3%.

**Figure 10:**
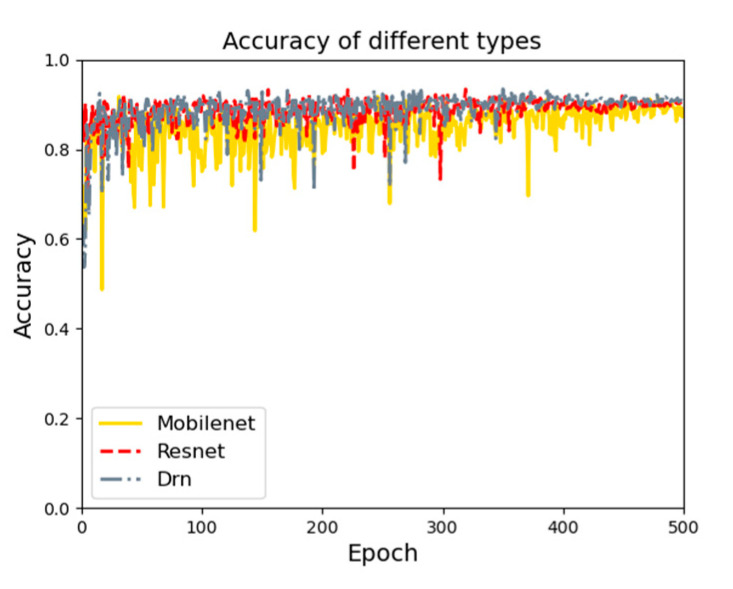
Types of feature extraction result curve.

With the same conditions, feature information of types (rocks and quicksand) is trained. The type of feature extraction result curve is shown in Figure 10. On the test set, three base networks are compared, and the Drn base network works best. The accuracy on the test set is 93.1%.

#### 4.3.2. MIOU and FWIOU

**Figure 11:**
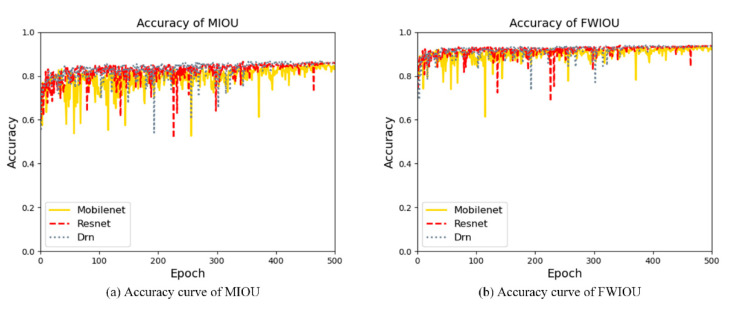
Accuracy of MIOU and FWIOU.

In feature extraction, there are some other indicators, such as MIOU (Mean Intersection Over Union) and FWIOU (Frequency Weighted Intersection Over Union). MIOU represents the ratio of intersection and union of predicted values. FWIOU stands for setting the weight according to the frequency of occurrence of each category. The accuracy of MIOU and FWIOU is shown in Figure 11a,b, respectively.

The accuracy rate of MIOU is 87.5% on the test set, and FWIOU is 93.4%. This also reflects the high efficiency of feature extraction.

#### 4.3.3. Hybrid Network for Scene Area Judgment

**Figure 12:**
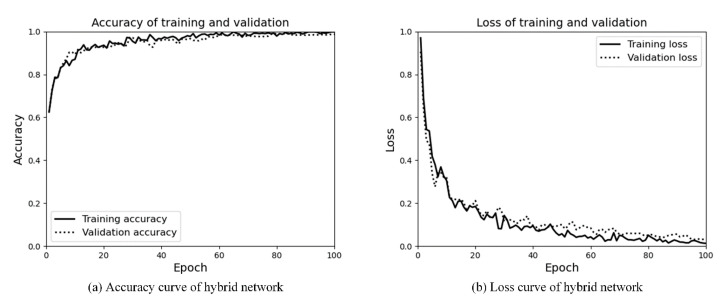
Accuracy and loss curves of the DeepLabV3+/Efficientnet hybrid network.

**Figure 13:**
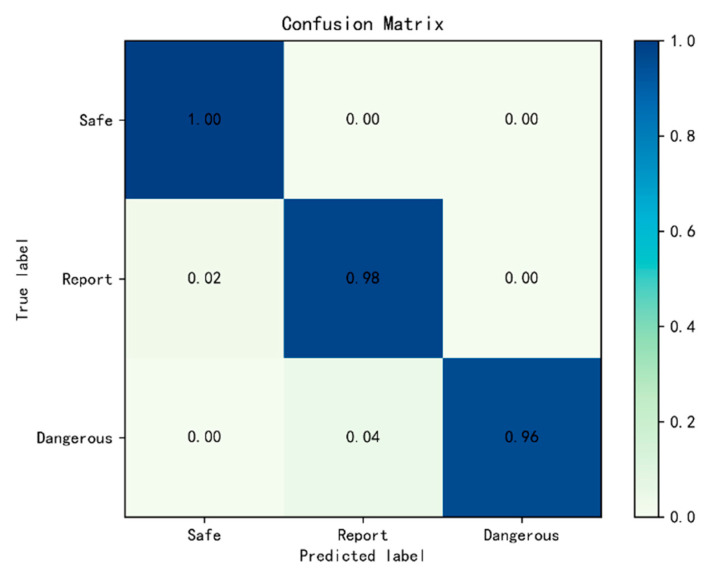
Confusion matrix of the DeepLabV3+/Efficientnet hybrid network for scene area judgment.

The DeepLabV3+/Efficientnet hybrid network is used to train the model and is tested on the test set. The accuracy of the training set is 99.8%, and that of the test set is 97.1%. The training results and loss curve are shown in Figure 12a,b. The model trains 100 epochs. From Figure 12a,b, it can be seen that, although the number of training times is small, the model converges rapidly, and the training set and the validation set have good effects. The confusion matrix of the test set is shown in Figure 13. From Figure 13, we can see that the possibility of a dangerous area being incorrectly judged as a report area is 4%, and the possibility of a report area being incorrectly judged as a safe area is 2%. In addition, we fully consider the impact of judgment errors in the most dangerous case and classify all of these 4% errors as judgment errors. Compared with the traditional method, the hybrid network method reduces the rate of judgment error better and proves the robustness of the hybrid network.

#### 4.3.4. Comparison

**Figure 14:**
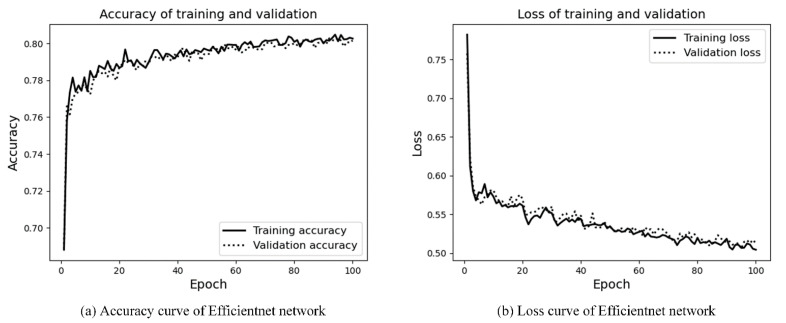
Accuracy and loss curves of Efficientnet.

The Efficientnet network is only used to judge the scene area. The experimental results are shown in Figure 14a,b. It can be seen that the number of training rounds is 100 epochs, the network converges quickly, and the accuracy of the training and validation is in good agreement. The trained model is performed on the test set, with an accuracy of 81% and a loss value of 0.51. Experimental results show that, compared with the Efficientnet network, the hybrid network is more effective.

## 5. Conclusions

In order to avoid the dangers of the Mars environment, the impact of road conditions on the Mars unmanned ground vehicle is considered. In this paper, the DeepLabV3+/Efficientnet hybrid network is proposed and is applied to the scene area judgment for the Mars unmanned vehicle system. This paper has three innovations: (1) the Mars unmanned vehicle system is conceived, with the impact of road conditions on the Mars unmanned ground vehicle solved; (2) an artificial intelligence algorithm is applied to the Mars unmanned vehicle system. The artificial intelligence algorithm improves the exploration accuracy of the Mars unmanned vehicle system; (3) the DeepLabV3+ network is used to extract features, with the problem of insufficient feature extraction capabilities of the Efficientnet network solved.

The DeepLabV3+/Efficientnet hybrid network has two advantages: (1) compared with the Efficientnet network, the accuracy of the hybrid network is improved by 18%; (2) compared with the Efficientnet network, the hybrid network can extract features better and has a smaller loss value. Experimental results show the effectiveness of the DeepLabV3+/Efficientnet hybrid network in the judgment of scene area, which ensures that the Mars unmanned vehicle system completes the Mars exploration mission.

## Figures and Tables

**Table 1:** Types of feature information.

Feature Information	Rock	Quicksand	Background
Type	2	1	0

**Table 2:** Image dataset.

Dataset	Training Set	Validation Set	Total
Number	480	130	610

**Table 3:** Dataset of data augmentation.

Data Augmentation	Training Set	Validation Set	Total
Number	4800	1300	6100

## Data Availability

Not applicable.
